# T2 MRI visible perivascular spaces in Parkinson’s disease: clinical significance and association with polysomnography measured sleep

**DOI:** 10.1093/sleep/zsae233

**Published:** 2024-10-08

**Authors:** Lena Meinhold, Antonio G Gennari, Heide Baumann-Vogel, Esther Werth, Simon J Schreiner, Christian Ineichen, Christian R Baumann, Ruth O’Gorman Tuura

**Affiliations:** Center for MR Research, University Children’s Hospital, Zurich, Switzerland; University of Zurich Sleep & Health Competence Center, Zurich, Switzerland; Center for MR Research, University Children’s Hospital, Zurich, Switzerland; Zentrum für Soziale Psychiatrie, Psychiatric University Hospital Zurich, Zurich, Switzerland; University of Zurich Sleep & Health Competence Center, Zurich, Switzerland; Department of Neurology, University Hospital, Zurich, Switzerland; University of Zurich Sleep & Health Competence Center, Zurich, Switzerland; Department of Neurology, University Hospital, Zurich, Switzerland; Department of Neurology, University Hospital, Zurich, Switzerland; University of Zurich Sleep & Health Competence Center, Zurich, Switzerland; Department of Neurology, University Hospital, Zurich, Switzerland; Center for MR Research, University Children’s Hospital, Zurich, Switzerland; University of Zurich Sleep & Health Competence Center, Zurich, Switzerland

**Keywords:** perivascular spaces, sleep, slow-wave sleep, glymphatic, waste clearance, neurodegeneration, Parkinson, magnetic resonance imaging

## Abstract

Poor sleep quality might contribute to the risk and progression of neurodegenerative disorders via deficient cerebral waste clearance functions during sleep. In this retrospective cross-sectional study, we explore the link between enlarged perivascular spaces (PVS), a putative marker of sleep-dependent glymphatic clearance, with sleep quality and motor symptoms in patients with Parkinson’s disease (PD). T2-weighted magnetic resonance imaging (MRI) images of 20 patients and 17 healthy control participants were estimated visually for PVS in the basal ganglia (BG) and centrum semiovale (CSO). The patient group additionally underwent a single-night polysomnography. Readouts included polysomnographic sleep features and slow-wave activity (SWA), a quantitative EEG marker of sleep depth. Associations between PVS counts, PD symptoms (MDS-UPDRS scores), and sleep parameters were evaluated using correlation and regression analyses. Intra- and inter-rater reproducibility was assessed with weighted Cohen`s kappa coefficient. BG and CSO PVS counts in both patients and controls did not differ significantly between groups. In patients, PVS in both brain regions was negatively associated with SWA (1–2 Hz; BG: *r*(15) = −.58, *p*_adj_ = .015 and CSO: *r*(15) = −.6, *p*_adj_ = .015). Basal ganglia PVS counts were positively associated with motor symptoms of daily living (IRR = 1.05, CI [1.01, 1.09], *p* = .007, *p*_adj_ = .026) and antidepressant use (IRR = 1.37, CI [1.05, 1.80], *p* = .021, *p*_adj_ = .043) after controlling for age. Centrum Semiovale PVS counts in patients were positively associated with a diagnosis of REM sleep behavior disorder (IRR = 1.39, CI [1.06, 1.84], *p* = .018, *p*_adj_ = .11). These results add to evidence that sleep deterioration may play a role in impairing glymphatic clearance via altered perivascular function, potentially contributing to disease severity in PD patients.

## Graphical Abstract

**Graphical Abstract:**
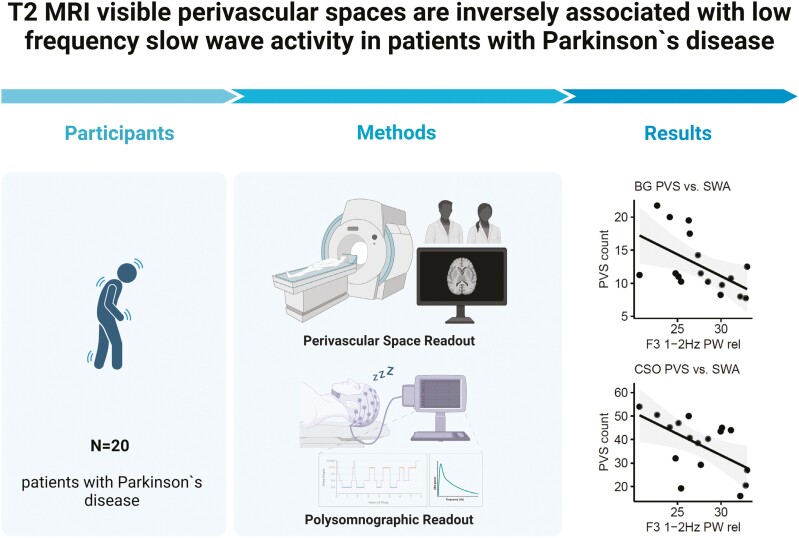

**Statement of Significance**

T2 MRI visible perivascular spaces (PVS) serve as biomarkers to investigate cerebral waste clearance functions, which are suspected to become dysfunctional in neurodegenerative disorders. Our study demonstrates, for the first time, that PVS, in Parkinson’s disease (PD) patients are associated with electroencephalographic low-frequency slow-wave activity (SWA), motor symptoms, and REM sleep behavior disorder diagnosis, providing evidence that sleep deterioration may impair cerebral waste clearance via perivascular mechanisms and potentially exacerbate disease severity in PD patients. Our findings highlight the importance of addressing sleep quality in PD management and offer new insights into the mechanisms underlying neurodegenerative disease progression.

Perivascular spaces (PVS) are fluid-filled spaces surrounding cerebral small vessels. They are important immunological sites [1] and form part of the brain`s metabolite clearance system [2]. This recently posited “glymphatic” system, is suggested to act as a macroscopic waste clearance mechanism for molecules of varying sizes through both tissue bulk flow and perivascular fluid flow, particularly during sleep [3]. Although there is debate about the precise anatomy of the PVS and the potential drainage routes of fluid from within the PVS, it is widely accepted that normal PVS function is important for brain health [4]. By contrast, enlargement of the PVS, which can be visually identified on T2-weighted MRI images in humans, is thought to reflect impaired PVS function and impaired neurofluid clearance [5].

Several factors can contribute to the enlargement of PVS, most notably aging [6], inflammation [7, 8], and vascular risk factors such as hypertension [9]. Consequently, enlarged PVS (EPVS) are thought to be clinically relevant and could be involved in the pathogenesis of neurodegenerative disorders [10, 11]. In Parkinson’s Disease (PD), the number of MRI visible PVS has been found to correlate with both cognitive [12, 13] as well as motor symptom severity [14], gray and white matter microstructural alterations [15], dopamine transporter availability [11] and CSF *α*-synuclein and t-tau levels [16]. Several neuroimaging studies have provided hints that waste clearance pathways could be impaired in Parkinson`s disease. Specifically, lower diffusion along PVS was identified in patients with PD, which correlated with the PVS impact as well as with symptom severity, using diffusion tensor imaging analysis along the perivascular space (DTI-ALPS) [17–21]. A low DTI-ALPS index, similar to T2-MRI visible PVS is thought to reflect glymphatic system impairment and has also been demonstrated in patients with Alzheimer’s disease [19, 22]. Other studies reported impaired drainage in meningeal lymphatic vessels using dynamic contrast-enhanced magnetic resonance imaging (DCE-MRI) [23] and altered CSF flow in the central aqueduct using phase-contrast MRI (PC-MRI) [24].

PD is a complex multifactorial neurodegenerative disease, characterized by an interplay of genetic predispositions and environmental factors, but mostly of unknown origin, hence idiopathic. Viral or bacterial infections, changes in the gut-brain axis, peripheral inflammation, and *α*-synuclein accumulation in the peripheral and subsequently central nervous system, accompanied by changes in neural circuits lead to severe neuroinflammation, oxidative stress, and neuronal degeneration in basal regions of the brain, creating a phenotype of motor symptoms (e.g. tremor, rigidity, and bradykinesia) and non-motor symptoms (e.g. cognitive decline, depression, and autonomic nervous system symptoms) [25]. Sleep disturbance is a highly common complaint of individuals with PD [26] and sleep quality is thought to underly both risk as well as disease progression in PD [27]. Glymphatic clearance is thought to rely on sleep [3] and sleep fragmentation or sleep loss often occurs already in the prodromal stage of PD [28, 29]. Therefore, glymphatic function may be impaired in individuals with PD [30], potentially contributing to the accumulation of proteins such as *α*-synuclein [31–33], as well as to the accumulation of immune cells and cytokines within the PVS, aggravating inflammation [30, 34, 35]. While an association between sleep, deficient glymphatic clearance, and disease severity in Alzheimer’s disease (AD) has received greater attention [36–38], this link remains underexplored in PD.

The purpose of this study was thus, to investigate the relationship between enlarged PVS, as a biomarker of impaired neurofluid clearance, and sleep parameters in patients with PD. If enlarged PVS indeed signifies impaired sleep-dependent clearance in PD, we expect to observe an inverse relationship between enlarged PVS and sleep quality, particularly SWA, as clearance is most efficient during periods of elevated delta power (SWA) [39]. Furthermore, we assessed whether motor and non-motor symptoms in patients with PD were associated with the PVS impacts.

## Methods

### Participants

The participant group of this retrospective cross-sectional study consisted of 20 patients with Parkinson`s disease (16 male, mean age 63 years) and 17 healthy controls (13 male, mean age 62 years) with no history of neurological or psychiatric illness. All participants were recruited from the Neurology Department of the University Hospital of Zurich, Switzerland between July 2014 and October 2018 and provided written informed consent to participate in a prospective study examining the link between MR imaging and spectroscopy measures, non-motor symptoms, and clinical outcome in PD [40]. Study methods and results are reported following the Strengthening the Reporting of Observational Studies in Epidemiology (STROBE) Statement for cross-sectional studies [41].

### Image acquisition and analysis

T2-weighted fast spin echo (FSE) MRI data were acquired with a 3T GE MR750 scanner, using an 8-channel head coil, with TE/TR = 109/5700 ms, FOV = 25.6 cm, acquisition matrix = 256 × 256, reconstruction matrix = 512 × 512, slice thickness = 2 mm. All MRI scans took place during the daytime. We visually inspected axial T2-weighted images and estimated PVS counts and severity scores in the basal ganglia (BG) and centrum semiovale (CSO), two brain regions where PVS are typically prominent, using a validated scoring system [42]. Although other deep gray matter regions e.g. midbrain/substantia nigra (SN), are interesting targets to study PVS in patients with PD, we aimed to ensure consistency with studies published previously and with the scoring system validated in REF [42]. Briefly, the procedure was as follows: Two independent blinded examiners visually determined PVS counts twice on a predefined axial slice (for the basal ganglia: the first slice above the anterior commissure; for the centrum semiovale: the first slice above the lateral ventricles). PVS on all sequences including T2 weighted images are defined as having signal intensity similar to that of CSF and appear round or ovoid in shape (with a diameter of up to 3 mm) when running perpendicular to the imaging plane, or linear in shape when running parallel to the imaging plane. Of the hemisphere with the higher count, we used the average count of both readers for subsequent statistical analyses. We determined lateralization of PVS based on the counts of the second readout, whenever both readers agreed. Using a cutoff of ≥2 counts for the BG and a cutoff of ≥4 counts for the CSO, participants were assigned to either left predominant, right predominant, or symmetric PVS impact.

### Symptom evaluation and laterality discrimination

PD symptoms were assessed in therapeutic ON condition using the Unified Parkinson’s Disease Rating Scale of the International Parkinson and Movement Disorders Society (MDS-UPDRS). Sum scores for the four sections (I: Non-motor aspects of daily living, II: Motor aspects of daily living, III: Motor examination, IV: Motor complications) were calculated. Lateralization of Motor symptoms was determined based on the MDS-UPDRS part III score: For each participant, items 20–26 were summed, yielding a total lateralized motor score for each side of the body. Using a cutoff of ≥2 points, patients were assigned to either the left predominant (PD-L), the right predominant (PD-R), or the symmetric (PD-S) group [43].

### Sleep measurements and analysis

18 patients additionally underwent single-night video-recorded polysomnography (PSG) in the sleep laboratory either some weeks before or after the MRI measurement (time difference between MRI and PSG dates: median = 3.36, IQR = 5.43 weeks). Exams were performed using an Embla N7000, RemLogic v3.2 system. PSGs were scored by experienced sleep experts based on the American Academy of Sleep Medicine (AASM) scoring guidelines [44]. Sleep parameters of interest were total sleep time (TST), percentage and absolute minutes of sleep stages N1, N2, N3, and REM, electroencephalographic (EEG) slow-wave activity (0.5–4 Hz and 1–2 Hz frequency bands), sleep efficiency (SE), sleep latency (SL), wake after sleep onset (WASO) and REM sleep abnormalities such as REM sleep without atonia, twitches, vocalization and video-graphically documented signs of acting out dreaming to increase the diagnostic certainty of REM sleep behavior disorder (RBD). EEG signal preprocessing included filtering (0.5 Hz high pass and 40 Hz low pass) as well as artifact removal. We performed EEG spectral analysis as previously described [45]. Briefly, we computed power spectra for whole night NREM sleep stages N2 + N3 and normalized average band power to 0.5–30 Hz power. We focused on SWA over frontal regions (F3, F4) and in the low-frequency range (1–2 Hz) because frontal SWA 1–2 Hz and slow oscillations relate to PD and markers of neurodegeneration more closely as compared to SWA in the higher delta range [45–48]. Sleep efficiency was calculated as total sleep time as a percentage of total time in bed; sleep latency was defined as absolute time [minutes] until the first appearance of N2 sleep; wake after sleep onset was defined as total time awake as a percentage of total time in bed after sleep onset. To control for sleep confounders, we evaluated patients’ clinical records for the presence of sleep disorders and calculated periodic limb movements (PLMS), apnea–hypopnea index (AHI), and arousal index (AI) from PSGs.

### Statistical analysis

Groupwise differences in PVS counts were evaluated with unpaired two-sample *t*-tests or Mann–Whitney U tests. Associations of PVS with Parkinson’s symptoms for each of the four sections of the MDS-UPDRS were evaluated with Pearson’s or Spearman’s correlation coefficients and with negative binomial regression models, covarying for age. Exploratory regression analyses were conducted for MDS-UPDRS single items if the entire scale showed an association with either BG or CSO PVS using negative binomial regression models with age as a covariate. Fisher’s exact tests were used, to determine whether patients with motor symptom asymmetry showed lateralization of PVS in the corresponding hemisphere. To test for the effects of sleep quality on PVS, negative binomial regression models with the covariates age and disease duration were used. Due to the known effects of antidepressants on sleep and the potential effects of depression (and antidepressants) on PVS [4] we tested for these effects using negative binomial regression models. Potential effects of sleep confounders (AHI, PLMS, and AI) were evaluated using correlation and regression analyses. For all regression models, covariates were selected based on biological plausibility and residuals were analyzed to check model assumptions. Results were corrected for multiplicity using the false discovery rate (FDR). Intra- and inter-rater reproducibility of the PVS counts and severity scores was assessed with weighted Cohen’s kappa coefficient. All statistical analyses were performed with RStudio, R version 4.3.2.; the two-sided significance level was predetermined at .05.

## Results

### Participants and PVS readout

**Figure 1.**
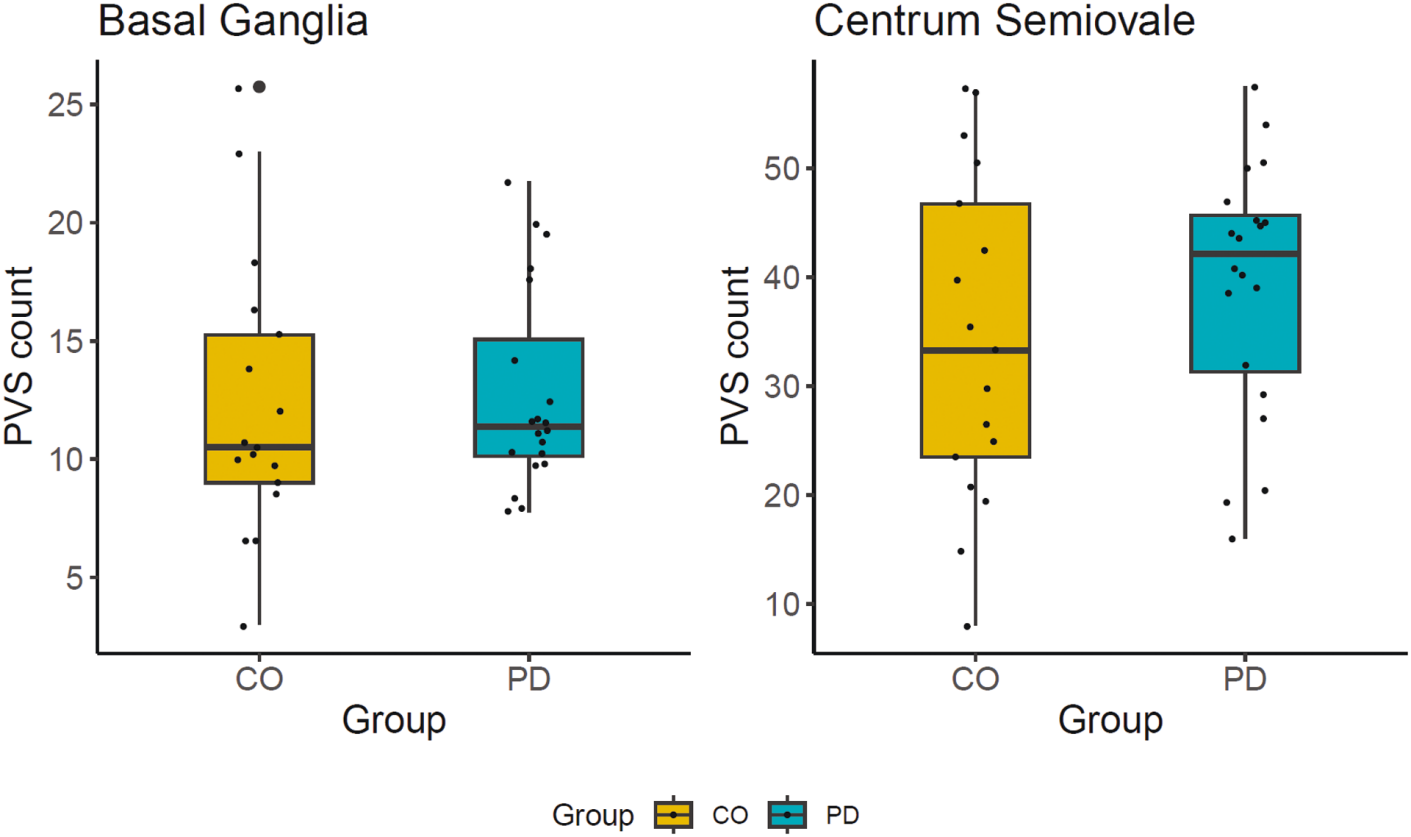
PVS counts in the basal ganglia and centrum semiovale in the patient- and control group. Counts were determined twice by two independent blinded examiners on a predefined axial slice in the basal ganglia and centrum semiovale respectively, yielding a total of four examinations. Boxplots show the average counts of both readers of all examinations.

Two patients did not have PSG measurements and were therefore excluded from all sleep analyses. Apart from one patient with periodic leg movements, none of the patients had clinically diagnosed sleep disorders, therefore the remaining 18 patients went into the sleep analyses. One patient was additionally excluded from the SWA analyses due to low signal quality. Two patients had unknown disease duration and were thus removed from all regression models in which disease duration was used as the covariate. Baseline characteristics as well as results of the PVS readout for patients and controls are shown in Table 1. The clinical characteristics of the PD group are shown in Table 2. (For an assessment of comorbidities, PD subtypes, laterality of motor symptoms, and medication see Supplementary Table S1A-B). PVS counts in both patients and controls increased with age but did not differ significantly between groups (Figure 1). Antidepressant use affected PVS in BG, but not CSO, when controlling for age (IRR = 1.37, CI [1.05, 1.80], *p* = .021, *p*_adj_ = .043). Analysis of sleep confounders showed that seven patients could qualify for sleep apnea (AHI > 5), two had elevated PLMS and 4 patients had an increased number of arousals. Neither of the indices were directly correlated with PVS. AHI significantly predicted CSO PVS in a multivariate regression model including age, PLMS, and AI; however, this effect did not survive FDR correction (IRR = 1.02, CI [1.00, 1.04], *p* = .040, *p*_adj_ = .093).

**Table 1.** Baseline Characteristics and Results of the Average PVS Readout

	CO	PD	SMD
**n**	17	20	
**Age (mean [SD])**	62.51 (12.83)	63.51 (6.61)	0.098
**BG PVS [count] (mean [SD])**	12.29 (5.91)	12.76 (4.25)	0.091
**CSO PVS [count] (mean [SD])**	34.31 (15.14)	39.20 (11.68)	0.362
**BG PVS [grade]**			0.487
** none/mild-grade**	7 (41.2)	5 (25.0)	
** moderate**	8 (47.1)	14 (70.0)	
** frequent**	2 (11.8)	1 (5.0)	
**CSO PVS [grade]**			0.594
** none/mild-grade**	1 (5.9)	0 (0.0)	
** moderate**	2 (11.8)	2 (10.0)	
** frequent**	8 (47.1)	6 (30.0)	
** severe**	6 (35.3)	12 (60.0)	

Mean values for patients (PD) and controls (CO) are given including standard mean difference (SMD). Note that according to STRIVE guidelines PVS counts are converted to severity scores in the following way: <10 = none/mild-grade, 11–20 = moderate, 21–40 = frequent and >40 = severe [42]. (BG: basal ganglia, CSO: centrum semiovale).

**Table 2.** Clinical Assessments in the Patient Group

	Overall	Missing (%)
*n*	*20*	
MDS-UPDRS I (mean [SD])	12.35 (4.65)	0
MDS-UPDRS II (mean [SD])	12.60 (3.70)	0
MDS-UPDRS III (mean [SD])	20.00 (7.52)	0
MDS-UPDRS IV (mean [SD])	6.85 (2.68)	0
MDS-UPDRS total (mean [SD])	51.80 (11.80)	0
Hoehn & Yahr Stage (mean [SD])	2.05 (0.43)	0
disease duration (mean [SD])	9.44 (4.20)	10
levodopa eq dose (mean [SD])	1090.28 (375.06)	10

Parkinson’s symptoms were assessed using the Unified Parkinson’s Disease Rating Scale of the International Parkinson and Movement Disorders Society (MDS-UPDRS, in therapeutic ON conditions), disease staging was performed using the modified Hoehn and Yahr Scale, disease duration was assessed as years since diagnosis, medication given as Levodopa-equivalent daily dose.

### Association with MDS-UPDRS

**Figure 2.**
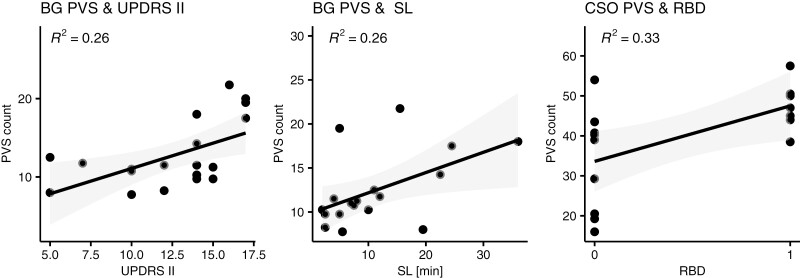
Relationship between (A) BG PVS counts and MDS-UPDRS II scores (B) BG PVS and sleep latency and (C) CSO PVS and a diagnosis of RBD. Shown are parameters that displayed direct correlation with PVS counts without FDR correction (uncorrected *p*-values: .023, .031, .021 for a, b, and c, respectively) Note that RBD = 1 corresponds to patients with a concomitant RBD diagnosis, RBD = 0 to patients without such diagnosis. Labels show the R², a straight line indicates linear fit with 95% confidence bounds.

In patients, basal ganglia PVS counts were positively correlated with MDS-UPDRS II scores, reflecting motor symptoms of daily living (*r*_s_(16) = .51, *p* = .023, *p*_adj_ = .12; Figure 2A), but this result did not survive FDR correction. Results of negative binomial regression models with age as a covariate showed a significant main effect of BG PVS on MDS-UPDRS II scores (*b* = 0.051, *p *= .007, *p*_adj_ = .026), in line with the previous result. Incidence rate ratios yielded that for every unit increase in the MDS-UPDRS II scale, BG PVS counts increased by 5%, (95% CI [1.01, 1.09]). Results of the exploratory regression analyses, investigating MDS-UPDRS II single items showed significant main effects of the items “speech” (b = 0.18, 95% CI [0.04, 0.31], *p* = .012), “chewing and swallowing” (b = 0.17, 95% CI [0.01, 0.32], *p* = .032) and “getting out of bed, a car, or a deep chair” (b = 0.23, 95% CI [0.03, 0.42], *p* = .023; Supplementary Figure S1). We found no evidence that patients with lateral motor symptom classification had more PVS in the contralateral hemisphere.

### Association with sleep

**Figure 3.**
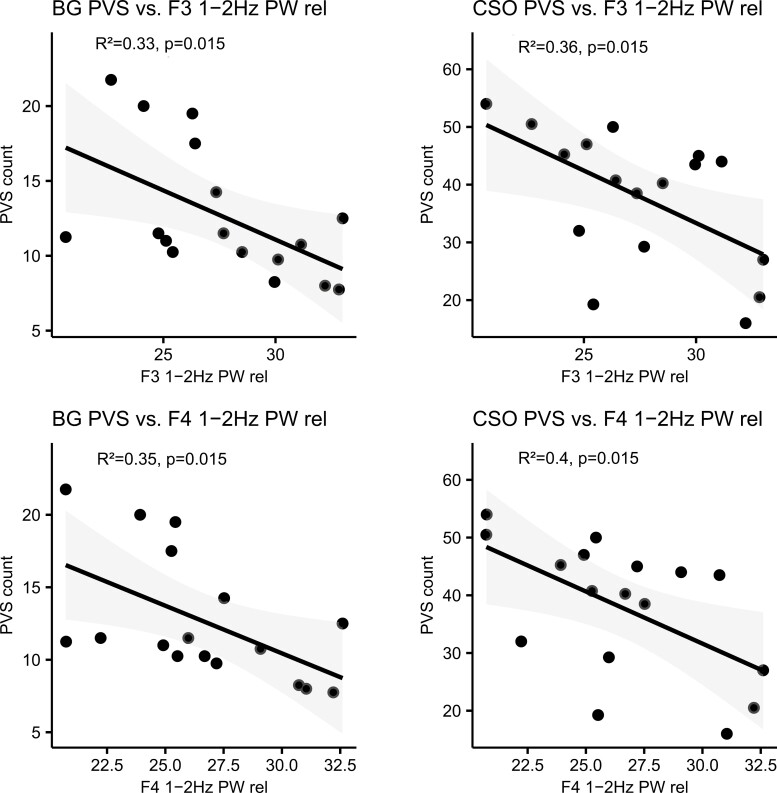
Direct correlation between PVS and relative 1–2 Hz slow-wave activity at F3 and F4. Slow-wave activity refers to the power spectra calculated for NREM sleep stages N2 + N3 and normalized to the 0.5–30 Hz power. Direct correlations are labeled with R² and FDR-adjusted *p*-values, a straight line indicates linear fit with 95% confidence bounds.

Mean values for sleep parameters as well as the calculated values for absolute and relative slow-wave activity in the patient group are shown in Supplementary Table S2. Both BG PVS and CSO PVS were correlated with slow-wave activity in the low delta frequency range (1–2 Hz) measured at frontal electrodes (for F3, BG PVS: *r*(15) = −.58, *p* = .015, *p*_adj_ = .015 and CSO PVS: *r*(15) = −.6, *p* = .013, *p*_adj_ = .015, see Figure 3 for full result). These associations remained significant when controlling for the effects of age and disease duration using negative binomial regression models (Table 3). Covarying for antidepressant use did not change this association (for F3, BG PVS: IRR = 0.94, CI [0.90, 0.99], *p*_adj_ = .012, CSO PVS: IRR = 0.95, CI [0.91, 0.98], *p*_adj_ = .004), neither did covarying for sleep confounders PLMS, AHI, and AI (Supplementary Table S3). We found no association of PVS with SWA in the whole delta frequency range (0.5–4 Hz; Supplementary Table S4). Analysis of sleep parameters demonstrated that BG PVS were positively correlated with sleep latency (*r*(16) = .51, *p* = .031, *p*_adj_ = .39), whereas CSO PVS were positively correlated with a diagnosis of REM sleep behavior disorder (*r*(16) = .57, *p* = .021, *p*_adj_ = .11; Figure 2B and C; however, these results did not hold after multiplicity correction. To control for the effects of age and disease duration, negative binomial regression models were calculated. Results showed that the association of BG PVS with SL was significant (b = 0.027, *p* = .013, IRR = 1.03, CI [1.01, 1.05], *p*_adj_ = .10), as well as the association of CSO PVS with RBD diagnosis (b = 0.33, *p* = .018, IRR = 1.39, CI [1.06, 1.84], *p*_adj_ = .14); however, both results did not survive multiplicity correction. Antidepressant use did not affect the relationship between CSO PVS and RBD, but the effect of sleep latency on BG PVS disappeared when antidepressant use was controlled for. We found no association of PVS with the other sleep quality measures (TST, N1, N2, N3, REM, SE, and WASO; Supplementary Tables S5, S6).

**Table 3.** Results of Negative Binomial Regression Models For PVS and Normalized Slow-Wave Activity From 1 to 2 Hz Frequency Range

	CSO PVS			BG PVS			CSO PVS			BG PVS		
*Predictors*	*IRR*	*95% CI*	*P*	*IRR*	*95% CI*	*P*	*IRR*	*95% CI*	*P*	*IRR*	*95% CI*	*P*
(Intercept)	20.20	4.55 to 89.23	**<.001**	19.21	2.53 to 143.40	**.004**	19.33	4.89 to 75.99	**<.001**	18.25	2.59 to 126.54	**.003**
age	1.03	1.01 to 1.05	**.002**	1.02	0.99 to 1.04	.153	1.03	1.01 to 1.05	**.001**	1.02	1.00 to 1.04	.117
disease duration	1.01	0.99 to 1.04	.306	1.00	0.96 to 1.04	.926	1.02	0.99 to 1.04	.274	1.00	0.96 to 1.04	.903
F3 1–2 Hz Pw rel	0.95	0.92 to 0.99	**.009**	0.94	0.90 to 0.99	**.013**						
F4 1–2 Hz Pw rel							0.95	0.92 to 0.98	**.006**	0.94	0.90 to 0.98	**.011**
Observations	16			16			16			16		
R^2^	0.802			0.632			0.840			0.672		

Separate regression models were run for slow-wave activity at F3 and F4 channels, respectively. All models include age and disease duration as covariates and show the FDR adjusted *p*-values for the predictors of interest (F3 1–2 Hz Pw rel and F4 1–2 Hz Pw rel). Significant *p*-values are shown in bold. (IRR, incidence rate ratio; BG, basal ganglia; CSO, centrum semiovale, F3 1–2 Hz Pw rel: relative 1–2 Hz spectral power measured at F3, F4 1–2 Hz Pw rel: relative 1–2 Hz spectral power measured at F4).

### Reproducibility

The inter-rater reproducibility of PVS counts for the first session was k = 0.6, (95% CI [0.4, 0.81]) for basal ganglia (BG) and k = 0.61, (CI [0.44, 0.79]) for centrum semiovale (CSO); for the second session it was k = 0.7, (CI [0.53, 0.86]) for basal ganglia (BG) and k = 0.61, (CI [0.41, 0.82]) for centrum semiovale (CSO). The intra-rater reproducibility for rater 1 was k = 0.72, (CI [0.49, 0.95]; BG) and k = 0.77, (CI [0.66, 0.88]; CSO); the intra-rater reproducibility for rater 2 was k = 0.48, (CI [0.28, 0.67]; BG) and k = 0.49, (CI [0.28, 0.69]; CSO). Reproducibility for the PVS severity ratings are given in Table 4.

**Table 4.** Quadratic Weighted Cohen’s Kappa Coefficients (k) Showing the Intra- and Inter-reader Agreement For PVS Counts and Severity Scores

	Kappa	
Agreement	PVS count	PVS severity
**Intra-reader**		
Rater 1—BG	0.72, CI [0.49, 0.95]	0.61, CI [0.37, 0.85]
Rater 1—CSO	0.77, CI [0.66, 0.88]	0.67, CI [0.5, 0.85]
Rater 2—BG	0.48, CI [0.28, 0.67]	0.3, CI [0.1, 0.49]
Rater 2—CSO	0.49, CI [0.28, 0.69]	0.43, CI [0.2, 0.67]
**Inter-reader**		
** **BG–S1	0.6, CI [0.4, 0.81]	0.49, CI [0.26, 0.73]
** **BG–S2	0.7, CI [0.53, 0.86]	0.49, CI [0.29, 0.7]
** **CSO–S1	0.61, CI [0.44, 0.79]	0.6, CI [0.4, 0.8]
** **CSO–S2	0.61, CI [0.41, 0.82]	0.6, CI [0.4, 0.8]

Note that the agreement is considered poor for k = 0.00–0.20, fair for k = 0.21–0.40, moderate for k = 0.41–0.6, good for k = 0.61–0.8 and very good for k = 0.81–1.00 according to Bland (2015) [49]. (BG: basal ganglia, CSO: centrum semiovale, S1: Session 1, S2: Session 2).

## Discussion

In this study, we investigated the association between T2 MRI visible PVS (during daytime) with motor symptom severity and sleep parameters assessed some weeks before or after MRI (median = 3.36, IQR = 5.43 weeks), in patients with Parkinson’s disease. Our results suggest that PVS are associated with motor symptoms in PD patients and are thus clinically relevant. Furthermore, our results provide evidence that the EPVS impact could be related to sleep quality, especially sleep depth (SWA).

### PVS readout

Although other studies identified differences in T2-visible PVS between PD patients and healthy age-matched controls [15, 50], we did not observe this difference in our sample. The patient group showed more T2 visible PVS in both white and gray matter compared to controls; however, the group comparison did not reach significance. Considering the comparatively small sample size, it is likely that the present study was underpowered to detect a true difference in PVS. Furthermore, considering that the sample consisted of patients in rather mild disease stages (mean MDS-UPDRS III = 28.33, mean LEDD = 1082.81, mean Hoehn and Yahr Stage = 2), it can be assumed that striatal neurodegeneration is less severe and a group difference would probably be more detectable in a sample of patients with more advanced PD, or potentially in brain regions that are earlier affected in PD, such as the substantia nigra (SN). However, enlarged PVS may not be a specific feature of PD. The natural, age-related functional alteration of the PVS could add to the general risk of developing idiopathic PD with aging [51] or PVS could appear secondary to pathological changes in the brains of patients, e.g. as an expression of neuroinflammation [4]. One study compared the total PVS volume fraction in individuals with familial and idiopathic PD with that of non-manifest carriers (NMC) and found that individuals with familial PD, particularly carriers of LRRK2 mutations, had the greatest increase in PVS impacts compared to NMCs [50], suggesting that a specific genetic background may contribute to the EPVS impact. In the present work, antidepressant use affected PVS in BG but not CSO; however, this effect may be part of the (vascular) depressive phenotype rather than an effect of antidepressant medication [52]. Furthermore, differences in PD subtypes and their associated pathophysiological changes could potentially also explain variation in PVS counts; however, our sample size would be inadequate to address this question.

### Clinical significance and motor symptoms

Previous studies have reported a link between enlarged basal ganglia PVS and vascular cognitive impairment [10], and symptom severity in PD [13–15]. The observed link between BG PVS and MDS-UPDRS II scores in the present study provides further evidence that impairment of motor functioning in daily living and potentially also cognitive skills may be worsened by deficits in neurofluid clearance. Consistent with our results, a recent 7T study showed a link between BG PVS number (but not volume) with the scores from part II of the MDS-UPDRS, after controlling for age, sex, and total brain volume [15]. The basal ganglia are heavily involved in motor functions and are one of the brain structures most severely affected by degenerative processes in PD patients [25]. Deficits in perivascular drainage in this region may therefore aggravate existing pathology; however, it remains uncertain whether they could contribute to the initial symptom onset. The same study also reported an association of BG PVS with the MDS-UPDRS sub-scores III and IV as well as the total score [15]; however, this association was absent in our sample. Again, considering the comparatively smaller sample size, it is likely that the present study was underpowered to detect such an association. In our exploratory regression analyses, the MDS-UPDRS II single items “speech,” “chewing and swallowing” and “getting out of bed, a car, or a deep chair” were associated with BG PVS. While the item “speech” appears also within the UPDRS III, the other two items are not assessed within the UPDRS III, suggesting that specific aspects of UPDRS II that are not captured within the UPDRS III could account for the observed associations. Interestingly, all of these tasks involve axial motor function and rely on motor networks involving the basal ganglia [53, 54] and structural changes in this brain region including enlarged PVS may therefore worsen these symptoms. Taken together, our findings suggest that basal ganglia PVS are clinically relevant and could indicate or even influence disease severity in PD patients. Centrum semiovale PVS on the contrary have been suggested to be associated with a clinical diagnosis of Alzheimer’s disease [10]. Correspondingly, we did not observe an association of CSO PVS with the MDS-UPDRS in our cohort; however, CSO PVS correlated with a diagnosis of RBD. Of note, a diagnosis of RBD may either reflect later stages of PD or the “body-first” subtype of PD with a higher global *α*-synuclein impact and faster cognitive decline [55], suggesting that CSO PVS may become more clinically relevant as the pathology spreads from the more basal regions of the brain to progressively more neocortical regions. Interestingly, a study by [56] reported a higher PVS impact (centrum semiovale, basal ganglia, substantia nigra, and brainstem PVS counts) in individuals who were diagnosed with idiopathic RBD compared to PD patients (with and without RBD). The autonomic pathology present in RBD patients as well as in the “body first” PD subtype, but not in the “brain first” PD subtype [55], could therefore be especially linked to deficient perivascular function, consistent with recent reports suggesting an autonomic contribution to cerebrospinal fluid dynamics in humans [57, 58].

### Sleep quality, sleep depth, and RBD

This study demonstrated for the first time, that BG and CSO PVS in patients with PD are inversely related to low-frequency SWA (1–2 Hz; Figure 3). Furthermore, this study provided evidence that RBD diagnosis may be related to CSO PVS, although this result weakened after multiplicity correction (Figure 2, Supplementary Table S6). Sleep disturbances in general, including difficulties with sleep initiation or maintenance, are common in patients with PD [59] and dopaminergic medications can negatively impact sleep quality [60]. Sleep problems in most patients initially emerge in the prodromal stage of PD as a result of early degenerative processes in brain structures that are involved in regulating sleep–wake states, such as the reticular formation and raphe nuclei [61]. A large population-based study found that poor sleep quality increased the risk of developing parkinsonism including PD 2 years after baseline measurement; however, this association disappeared with longer follow-up times, confirming sleep disturbances to be a biomarker for prodromal PD [29]. Interestingly, recent neuroimaging studies have found a link between poor sleep efficiency and enlarged BG PVS [62], as well as a link between enlarged CSO PVS and lighter sleep (longer N1 sleep and shorter N3 sleep duration) in healthy older adults [63]. In patients with cerebrovascular disease, BG PVS were shown to correlate with poor sleep efficiency, longer WASO, and shorter N3 duration [64]. Taken together, these studies and our results suggest that the link between PVS and sleep quality is not unique to PD patients, but sleep disturbance and PVS severity may still aggravate the neurodegenerative process. In the present sample, many patients displayed high values of wake after sleep onset, but surprisingly this parameter was not associated with PVS counts, nor was the duration of N3 stage sleep. However, we identified strong associations of perivascular space counts with slow-wave activity in the low delta frequency range (1–2 Hz). Therefore, rather than the total time spent in deep NREM sleep, the intensity of slow oscillatory brain activity in the 1–2 Hz frequency bands during NREM sleep may be more critical for perivascular functioning. A similar association between the EPVS impact and SWA (0.5–4 Hz) was also demonstrated in patients with arteriosclerotic cerebral small vessel disease [64]. Interestingly, lower SWA has also been shown to relate to motor deterioration, predominantly of axial motor symptoms in PD patients [65, 66]. Together with our identified associations between the EPVS impacts with both axial motor symptoms and SWA, these findings could indicate that patients progress faster owing to reduced sleep-dependent clearance [39]. While we did not evaluate the overnight decline in SWA as a measure for homeostatic sleep pressure, it is noteworthy to contemplate that diminished homeostatic sleep pressure, as a common cause for extended sleep onset latency and decreased SWA might also contribute to the observed link between PVS, motor impairment and sleep quality. Taken together, the results of the present study add evidence that sleep quality may alter perivascular morphology and—along with it—drainage function and could contribute to disease severity in patients with PD. However, they also demonstrate that the link between PVS and sleep warrants further investigation in larger patient cohorts.

### Cerebral waste clearance dysfunction in PD?

Given the associations of T2-MRI visible PVS with motor symptoms and sleep parameters in our and other studies, it is particularly interesting to consider the potential role of neurofluid dynamics in PD risk and progression. Several neuroimaging studies have provided hints that waste clearance pathways could be impaired in PD [17–21, 23, 24]. Neurofluid dynamics likely rely on cerebrovascular activity [58], and on slow oscillatory brain activity during NREM sleep to drive perivascular fluid motion [67, 68]. Not only do patients with PD show reduced EEG spectral power in the delta frequency range [69, 70] but they also show a decoupling between the fMRI global signal and CSF flow [71], seen during sleep in the healthy brain [72], indicating either a disruption in the neurovascular unit or a blunted response of CSF dynamics to cerebrovascular activity in PD. Consistent with this idea, evidence from neuroimaging studies suggests that alterations in neurovascular coupling may be part of the neurobiological changes seen in patients with PD [73, 74]. Evidence from rodent studies shows that the highest coherence between neural activity and hemodynamic signals is found during NREM sleep [75] when slow large oscillations in the diameter of pial arteries and penetrating arterioles occur, inducing dynamic changes in the volume of the PVS [76]. The specific role that slow-wave activity could play in the clearance of aggregation-prone proteins is further highlighted by a study showing that pharmacological sleep slow-wave enhancement with sodium oxybate reduces *α*-synuclein deposition in mouse models of Parkinson’s disease [77]. The latter and other rodent studies also suggest that alterations in the functioning of aquaporin-4 water channels, which are key modulators of the glymphatic pathway, could be implicated in PD disease progression [31, 33]. Taken together, altered neurofluid dynamics could play a role in the pathophysiology of PD and may be aggravated by sleep disturbances, particularly reduced low-frequency SWA, in patients.

Apart from the putative neuroprotective effects of the brain’s waste clearance system, a number of other mechanisms are plausible, that could account for an association between sleep quality and PD symptoms and disease progression, e.g. via the plasticity function of sleep [78] and intimate connection with the immune- and stress systems [79, 80]. Since these processes probably act in concert with the neurofluid drainage function, determining their relative importance might further inform our understanding of the role of sleep in the pathophysiology of Parkinson’s disease.

### Limitations

This study had several limitations. Firstly, the patient group had a limited sample size, and some of the results regarding sleep quality and clinical parameters did not survive a multiple comparisons correction. Since the hypotheses for these findings were prespecified, we report both FDR-corrected and uncorrected results but advise the reader to interpret results that did not survive correction with caution. Secondly, due to the retrospective analysis of the PSG data, PSGs were not recorded in the control group limiting the conclusions regarding the influence of sleep on the neurodegenerative process. Thus, the present study was not able to test whether the associations between PVS and sleep parameters were specific to PD symptomatology, but other studies have reported such links in healthy older adults [62, 63]. Moreover, there were time differences of several weeks between the PSG recordings and the MRI measurements and the latter took place during daytime. Although we do not suspect substantial changes in PVS counts within such short timeframes, the missing temporal link between the two assessments may be considered a limitation. The moderate inter- and intra-rater reproducibility of the PVS readout illustrates a limitation of the manual PVS assessment method, but automated methods for estimating the number of perivascular spaces may offer improved reliability for the assessment of PVS severity.

## Conclusion

In the present study, PVS in both BG and CSO were negatively associated with low-frequency slow-wave activity (1–2 Hz). Additionally, more PVS in the basal ganglia were associated with impaired motor symptoms and antidepressant use in patients with PD, while more PVS in white matter were weakly associated with a diagnosis of REM sleep behavior disorder. Sleep deterioration in patients with PD is likely to interact with the biological effects of aging as well as other disease-specific processes in the brain, which potentially involve a progressive decline in the function of the glymphatic system. Interventions targeted at improving sleep quality in patients with PD could therefore offer an opportunity to positively influence motor functions and may be able to slow disease progression, via the aforementioned mechanisms, including neurofluid clearance [66].

## Supplementary material

Supplementary material is available at *SLEEP* online.

zsae233_suppl_Supplementary_Tables_S1-S6_Figures_S1

## Data Availability

The data underlying this article can be shared on reasonable request to the corresponding author, with permission from the relevant ethics committee.
