# Supplementary material for: T2 MRI visible perivascular spaces in Parkinson’s disease: clinical significance and association with polysomnography measured sleep
Source: Sleep. 2024 Oct 8;48(1):zsae233. doi: 10.1093/sleep/zsae233 (PMC11725513; doi:10.1093/sleep/zsae233)
Supplement: zsae233_suppl_Supplementary_Tables_S1-S6_Figures_S1 [file zsae233_suppl_supplementary_tables_s1-s6_figures_s1.docx]

**T2 MRI visible Perivascular Spaces in Parkinson`s Disease: clinical significance and association with polysomnography measured sleep.**

Lena Meinhold^1,2^, Antonio G. Gennari^1^, Heide Baumann-Vogel^3^, Esther Werth^2,4^, Simon J. Schreiner^2,4^, Christian Ineichen^4^, Christian R. Baumann^2,4^, Ruth O'Gorman Tuura^1,2^

^1^Center for MR Research, University Children’s Hospital, Zurich, Switzerland

^2^University of Zurich Sleep & Health Competence Center

^3^Psychiatric University Hospital Zurich, Zentrum für Soziale Psychiatrie

^4^Department of Neurology, University Hospital, Zurich, Switzerland

**Corresponding author:** Lena Meinhold, Lenggstrasse 30, 8008 Zurich, Switzerland, [Lena.Meinhold@kispi.uzh.ch](mailto:Lena.Meinhold@kispi.uzh.ch)

**Authors Contact Details**

Lena Meinhold: [Lena.Meinhold@kispi.uzh.ch](mailto:Lena.Meinhold@kispi.uzh.ch), ORCID: 0009-0001-9000-2733

Antonio G. Gennari: [AntonioGiulio.Gennari@kispi.uzh.ch](mailto:AntonioGiulio.Gennari@kispi.uzh.ch), ORCID: 0000-0003-2224-0083

Heide Baumann-Vogel: [Heide.Vogel@gmx.de](mailto:Heide.Vogel@gmx.de), ORCID: 0009-0003-2415-3618

Esther Werth: [Esther.Werth.p1@gmail.com](mailto:Esther.Werth.p1@gmail.com), ORCID: 0000-0002-5099-3122

Simon Schreiner: [Simon.Schreiner@usz.ch](mailto:Simon.Schreiner@usz.ch), ORCID: 0000-0002-5150-6066

Christian Ineichen: [Christian.Ineichen@usz.ch](mailto:Christian.Ineichen@usz.ch), ORCID: 0000-0002-6087-0644

Christian Baumann: [Christian.Baumann@usz.ch](mailto:Christian.Baumann@usz.ch), ORCID: 0000-0003-3417-1978

Ruth O'Gorman Tuura: [Ruth.Tuura@kispi.uzh.ch](mailto:Ruth.Tuura@kispi.uzh.ch), ORCID: 0000-0001-5932-7786

**Supplement**

**Table S1a:** **Assessment of comorbidities, Parkinson`s disease subtype and laterality of motor symptoms in the patient group.**

| **comorbidities** | **frequency** |
| --- | --- |
| urinary symptoms | 9 |
| constipation | 7 |
| depression or depressive symptoms | 5 |
| orthosthatic dysregulation / vertigo | 5 |
| hyposmia | 3 |
| sexual dysfunction | 2 |
| hyperhidrosis | 2 |
| pain | 2 |
| hypersalivation | 2 |
| hypercholesterinemia | 1 |
| hypothyroidism | 1 |
| spondylodesis | 1 |
| pulmonary embolism | 1 |
| cataract surgery | 1 |
| sensory polyneuropathy | 1 |
| cervicocephalic and lumbospondylogenic syndrome | 1 |
| implantable cardioverter defibrillator | 1 |
| restless-legs-syndrome | 1 |
| mnestic disturbances | 1 |
| hyposexuality | 1 |
| recurrent panic attacks | 1 |
| **subtype (%)** |  |
| akinetic-rigid | 10 (52.4) |
| equivalent | 4 (19.0) |
| tremor dominant | 5 (23.8) |
| NA | 1 (4.8) |
| **laterality of motor symptoms (%)** |  |
| PD-L | 11 (55.6) |
| PD-R | 5 (27.8) |
| PD-S | 4 (16.7) |

Comorbidities were assessed to exclude the presence of sleep disorders. Apart from one patient with restless-legs-syndrome, no other sleep disorders were present. Parkison`s disease subtype was determined based on a classification into akinetic-rigid, tremor-dominant or equivalent type. Laterality of motor symptoms was determined based on the MDS-UPDRS part III score, as described in Poletti et al., 2013.

**Table S1b: Use of medication in the patient group.**

| **medication (n = 18)** | **frequency** |
| --- | --- |
| **antiparkinsonian agents** | 18 |
| **cardiovascular agents** | 8 |
| **antidepressants** | 7 |
| SSRI | 5 |
| SARI | 1 |
| SNRI | 1 |
| MAO-I | 1 |
|  |  |
| **dietary or herbal supplements** | 7 |
| **proton pump inhibitors** | 4 |
| **anti-inflammatory agents** | 3 |
| **hypnotics** | 2 |
| **chondroprotective agents** | 2 |
| **muscle relaxants** | 1 |
| **opioid analgesics** | 1 |
| **antimuscarinic** | 2 |
| **alpha-1 adrenergic blockers** | 1 |
| **anticonvulsants** | 1 |
| **thyroid hormone replacement** | 1 |
| **combination inhalers** | 1 |
| **contraceptives** | 1 |

Detailed documentation of medication was available in 18 of 20 patients and was evaluated due to the known effects of antidepressants on sleep, as well as the known effects of depression (and potentially antidepressant use) on perivascular spaces. Data showed that 7 patients used antidepressant medication, predominantly SSRIs. (SSRI: selective serotonin reuptake inhibitor, SARI: serotonin antagonist and reuptake inhibitor, SNRI: serotonin and norepinephrine reuptake inhibitor, MAO-I: monoamine oxidase inhibitor)

**Table S2: Mean values for sleep parameters as well as values for absolute and relative slow wave activity in the patient group.**

|  | **Overall** | **Missing (%)** |
| --- | --- | --- |
| **n** | 18 |  |
| **TST [min] (mean (SD))** | 359.84 (47.65) | 0 |
| **REM [min] (mean (SD))** | 57.24 (24.93) | 0 |
| **N1 [min] (mean (SD))** | 56.08 (32.60) | 0 |
| **N2 [min] (mean (SD))** | 204.13 (58.84) | 0 |
| **N3 [min] (mean (SD))** | 42.39 (36.49) | 0 |
| **SL [min] (mean (SD))** | 11.11 (9.23) | 0 |
| **SE [%] (mean (SD))** | 82.94 (10.13) | 0 |
| **WASO [min] (mean (SD))** | 17.06 (10.13) | 0 |
| **RBD (%)** |  | 11.1 |
| **no** | 9 (50.0) |  |
| **yes** | 7 (38.9) |  |
| **NA** | 2 (11.1) |  |
|  |  |  |
| **AHI (mean (SD))** | 5.59 (6.17) | 0 |
| **PLMS (mean (SD))** | 4.52 (9.09) | 0 |
| **Arousal Index (mean (SD))** | 10.43 (7.02) | 0 |
|  |  |  |
| **F3 1-2Hz Pw abs [W/Hz] (mean (SD))** | 66.61 (27.15) | 5.6 |
| **F3 1-2Hz Pw rel (mean (SD))** | 27.55 (3.62) | 5.6 |
| **F4 1-2Hz Pw abs [W/Hz] (mean (SD))** | 68.15 (35.20) | 5.6 |
| **F4 1-2Hz Pw rel (mean (SD))** | 26.58 (3.66) | 5.6 |
| **F3 0.5-4Hz Pw abs [W/Hz] (mean (SD))** | 61.08 (22.40) | 5.6 |
| **F3 0.5-4Hz Pw rel (mean (SD))** | 80.83 (4.75) | 5.6 |
| **F4 0.5-4Hz Pw abs [W/Hz] (mean (SD))** | 63.96 (31.28) | 5.6 |
| **F4 0.5-4Hz Pw rel (mean (SD))** | 79.62 (5.49) | 5.6 |

Slow wave activity (SWA) refers to the power spectra calculated for NREM sleep stages N2 + N3, for the relative SWA, the values of the absolute SWA were normalized to the 0.5-30 Hz power. (TST: total sleep time, REM: rapid eye movement sleep, SL: sleep latency, SE: sleep efficiency, WASO: wake after sleep onset, RBD: REM sleep behaviour disorder diagnosis, AHI: apnea-hypopnea index, PLMS: periodic limb movements, AI: arousal index)

**Figure S1 – Scatter plots for basal ganglia PVS versus single items of the MDS-UPDRS II.**


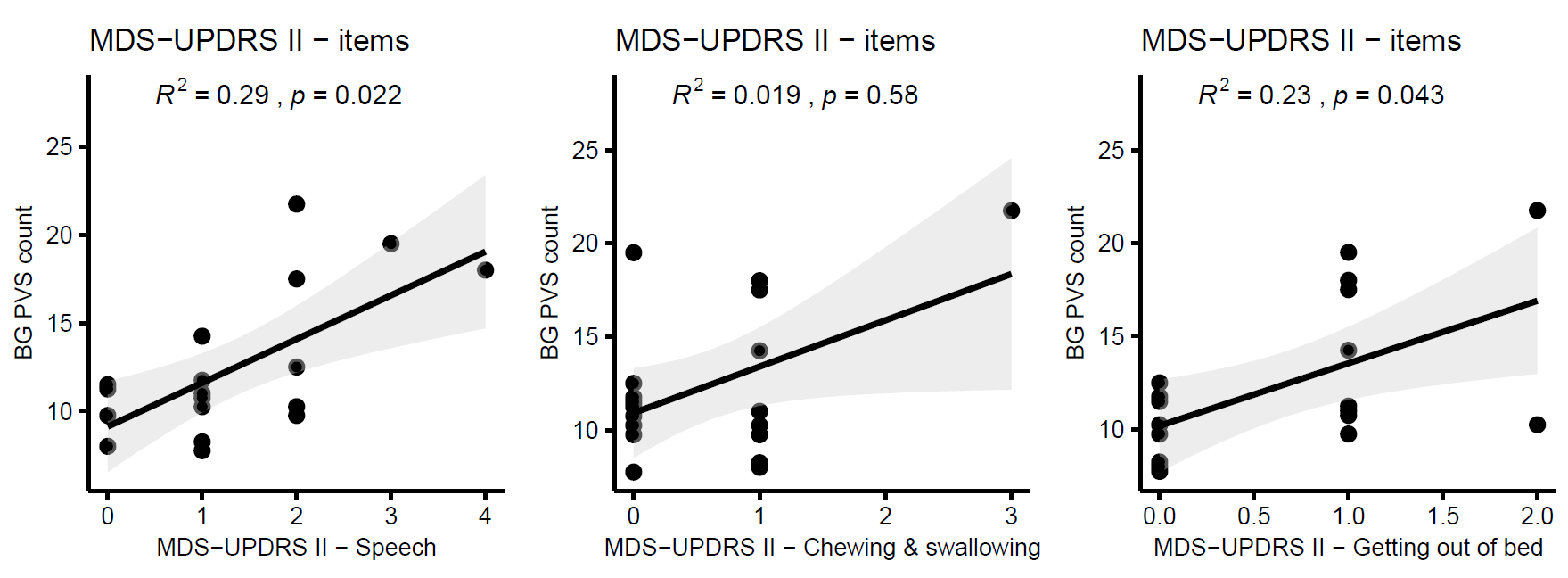


Regression analyses showed significant main effects of the single items “speech” (b = 0.18, 95% CI [ 0.04, 0.31], p = 0.012), “chewing and swallowing” (b = 0.17, 95% CI [ 0.01, 0.32], p = 0.032) and “getting out of bed, a car, or a deep chair” (b = 0.23, 95% CI [ 0.03, 0.42], p = 0.023) when controlling for the effect of age. Labels are showing the age uncorrected correlations with R² and corresponding p-value.

**Table S3: Results of negative binomial regression models for Basal Ganglia PVS and sleep parameters.**

|  | **BG PVS** | | | **BG PVS** | | | **BG PVS** | | | **BG PVS** | | |
| --- | --- | --- | --- | --- | --- | --- | --- | --- | --- | --- | --- | --- |
| *Predictors* | *IRR* | *95% CI* | *p* | *IRR* | *95% CI* | *p* | *IRR* | *95% CI* | *p* | *IRR* | *95% CI* | *p* |
| (Intercept) | 4.75 | 0.93 – 23.84 | 0.060 | 5.19 | 1.01 – 26.09 | **0.047** | 2.13 | 0.14 – 31.74 | 0.583 | 4.22 | 0.85 – 20.43 | 0.077 |
| Age | 1.02 | 0.99 – 1.05 | 0.281 | 1.02 | 0.99 – 1.05 | 0.160 | 1.03 | 0.99 – 1.07 | 0.183 | 1.02 | 0.99 – 1.04 | 0.205 |
| disease duration | 0.99 | 0.95 – 1.03 | 0.590 | 0.98 | 0.95 – 1.02 | 0.440 | 0.98 | 0.94 – 1.02 | 0.418 | 0.99 | 0.95 – 1.03 | 0.608 |
| N1 [min] | 1.00 | 0.99 – 1.01 | 0.801 |  |  |  |  |  |  |  |  |  |
| N2 [min] |  |  |  | 1.00 | 1.00 – 1.00 | 0.552 |  |  |  |  |  |  |
| N3 [min] |  |  |  |  |  |  | 1.00 | 0.99 – 1.01 | 0.504 |  |  |  |
| REM [min] |  |  |  |  |  |  |  |  |  | 1.00 | 1.00 – 1.01 | 0.651 |
| N | 16 | | | 16 | | | 16 | | | 16 | | |
| R^2^ | 0.167 | | | 0.193 | | | 0.200 | | | 0.179 | | |

|  | **BG PVS** | | | **BG PVS** | | | **BG PVS** | | | **BG PVS** | | |
| --- | --- | --- | --- | --- | --- | --- | --- | --- | --- | --- | --- | --- |
| *Predictors* | *IRR* | *95% CI* | *p* | *IRR* | *95% CI* | *p* | *IRR* | *95% CI* | *p* | *IRR* | *95% CI* | *p* |
| (Intercept) | 1.57 | 0.26 – 8.76 | 0.614 | 1.98 | 0.20 – 19.20 | 0.558 | 4.65 | 0.99 – 21.12 | **0.049** | 7.88 | 1.59 – 38.73 | **0.011** |
| Age | 1.03 | 1.00 – 1.06 | **0.032** | 1.02 | 0.99 – 1.04 | 0.175 | 1.02 | 0.99 – 1.04 | 0.175 | 1.01 | 0.98 – 1.03 | 0.558 |
| disease duration | 0.99 | 0.96 – 1.03 | 0.670 | 1.00 | 0.96 – 1.05 | 0.992 | 1.00 | 0.96 – 1.05 | 0.992 | 0.98 | 0.94 – 1.01 | 0.190 |
| SL [min] | 1.03 | 1.01 – 1.05 | **0.013** |  |  |  |  |  |  |  |  |  |
| SE [%] |  |  |  | 1.01 | 0.99 – 1.03 | 0.339 |  |  |  |  |  |  |
| WASO [min] |  |  |  |  |  |  | 0.99 | 0.97 – 1.01 | 0.339 |  |  |  |
| RBD |  |  |  |  |  |  |  |  |  | 1.42 | 1.01 – 2.00 | **0.045** |
| N | 16 | | | 16 | | | 16 | | | 16 | | |
| R^2^ | 0.572 | | | 0.239 | | | 0.239 | | | 0.457 | | |

Separate regression models were run for all sleep parameters including age and disease duration as covariates. (IRR: incidence rate ratio, BG: basal ganglia, CSO: centrum semiovale, SL: sleep latency, SE: sleep efficiency, RBD: REM sleep behaviour disorder).

**Table S4: Results of negative binomial regression models for Centrum Semiovale PVS and sleep parameters.**

|  | **CSO PVS** | | | **CSO PVS** | | | **CSO PVS** | | | **CSO PVS** | | |
| --- | --- | --- | --- | --- | --- | --- | --- | --- | --- | --- | --- | --- |
| *Predictors* | *IRR* | *95% CI* | *p* | *IRR* | *95% CI* | *p* | *IRR* | *95% CI* | *p* | *IRR* | *95% CI* | *p* |
| (Intercept) | 6.25 | 1.44 – 27.09 | **0.014** | 7.26 | 1.68 – 31.28 | **0.009** | 1.51 | 0.12 – 18.79 | 0.731 | 5.50 | 1.43 – 21.07 | **0.013** |
| Age | 1.03 | 1.01 – 1.06 | **0.016** | 1.03 | 1.01 – 1.06 | **0.014** | 1.05 | 1.01 – 1.10 | **0.006** | 1.03 | 1.01 – 1.05 | **0.012** |
| disease duration | 0.99 | 0.96 – 1.02 | 0.581 | 0.99 | 0.95 – 1.03 | 0.526 | 0.98 | 0.95 – 1.02 | 0.284 | 0.99 | 0.96 – 1.03 | 0.695 |
| N1 [min] | 1.00 | 0.99 – 1.00 | 0.675 |  |  |  |  |  |  |  |  |  |
| N2 [min] |  |  |  | 1.00 | 1.00 – 1.00 | 0.727 |  |  |  |  |  |  |
| N3 [min] |  |  |  |  |  |  | 1.00 | 1.00 – 1.01 | 0.145 |  |  |  |
| REM [min] |  |  |  |  |  |  |  |  |  | 1.00 | 1.00 – 1.01 | 0.118 |
| N | 16 | | | 16 | | | 16 | | | 16 | | |
| R^2^ | 0.424 | | | 0.421 | | | 0.523 | | | 0.552 | | |

|  | **CSO PVS** | | | **CSO PVS** | | | **CSO PVS** | | | **CSO PVS** | | |
| --- | --- | --- | --- | --- | --- | --- | --- | --- | --- | --- | --- | --- |
| *Predictors* | *IRR* | *95% CI* | *p* | *IRR* | *95% CI* | *p* | *IRR* | *95% CI* | *p* | *IRR* | *95% CI* | *p* |
| (Intercept) | 6.33 | 1.35 – 29.92 | **0.024** | 3.51 | 0.43 – 28.32 | 0.232 | 6.77 | 1.67 – 27.40 | **0.008** | 11.03 | 3.08 – 39.34 | **<0.001** |
| Age | 1.03 | 1.01 – 1.06 | **0.018** | 1.03 | 1.01 – 1.05 | **0.012** | 1.03 | 1.01 – 1.05 | **0.012** | 1.02 | 1.00 – 1.04 | 0.051 |
| disease duration | 0.99 | 0.96 – 1.03 | 0.605 | 1.00 | 0.96 – 1.04 | 0.985 | 1.00 | 0.96 – 1.04 | 0.985 | 0.98 | 0.95 – 1.01 | 0.127 |
| SL [min] | 1.00 | 0.98 – 1.02 | 0.828 |  |  |  |  |  |  |  |  |  |
| SE [%] |  |  |  | 1.01 | 0.99 – 1.02 | 0.416 |  |  |  |  |  |  |
| WASO [min] |  |  |  |  |  |  | 0.99 | 0.98 – 1.01 | 0.416 |  |  |  |
| RBD |  |  |  |  |  |  |  |  |  | 1.39 | 1.06 – 1.84 | **0.018** |
| N | 16 | | | 16 | | | 16 | | | 16 | | |
| R^2^ | 0.416 | | | 0.456 | | | 0.456 | | | 0.680 | | |

Separate regression models were run for all sleep parameters including age and disease duration as covariates. (IRR: incidence rate ratio, BG: basal ganglia, CSO: centrum semiovale, SL: sleep latency, SE: sleep efficiency, RBD: REM sleep behaviour disorder).

**Table S5. Results of negative binomial regression models for PVS and normalized slow wave activity from 0.5-4Hz frequency range.**

|  | **CSO PVS** | | | **BG PVS** | | | **CSO PVS** | | | **BG PVS** | | |
| --- | --- | --- | --- | --- | --- | --- | --- | --- | --- | --- | --- | --- |
| *Predictors* | *IRR* | *95% CI* | *p* | *IRR* | *95% CI* | *p* | *IRR* | *95% CI* | *p* | *IRR* | *95% CI* | *p* |
| (Intercept) | 20.04 | 1.19 – 337.07 | **0.044** | 0.46 | 0.02 – 12.64 | 0.646 | 64.74 | 2.38 – 1788.76 | **0.015** | 4.66 | 0.05 – 373.46 | 0.499 |
| Age | 1.03 | 1.01 – 1.05 | **0.006** | 1.03 | 1.00 – 1.05 | **0.038** | 1.03 | 1.00 – 1.05 | **0.029** | 1.02 | 0.99 – 1.05 | 0.139 |
| disease duration | 1.01 | 0.97 – 1.04 | 0.661 | 0.97 | 0.94 – 1.01 | 0.197 | 1.03 | 0.98 – 1.07 | 0.233 | 0.99 | 0.93 – 1.04 | 0.648 |
| F3 0.5-4Hz Pw | 0.98 | 0.95 – 1.01 | 0.278 | 1.02 | 0.99 – 1.06 | 0.181 |  |  |  |  |  |  |
| F4 0.5-4Hz Pw |  |  |  |  |  |  | 0.97 | 0.94 – 1.01 | 0.100 | 1.00 | 0.95 – 1.05 | 0.889 |
| Observations | 16 | | | 16 | | | 16 | | | 16 | | |
| R^2^ | 0.574 | | | 0.399 | | | 0.643 | | | 0.269 | | |

Separate regression models were run for slow wave activity at F3 and F4 channels respectively. All models include age and disease duration as covariates. (IRR: incidence rate ratio, BG: basal ganglia, CSO: centrum semiovale, F3 0.5-4Hz Pw: relative 0.5-4Hz spectral power measured at F3, F4 0.5-4Hz Pw: relative 0.5-4Hz spectral power measured at F4).

**Hoehn & Yahr Staging:**

0: No signs of disease, 1: Unilateral disease, 1.5: Unilateral plus axial involvement, 2: Bilateral disease, without impairment of balance, 2.5: Mild bilateral disease, with recovery on pull test. 3: Mild to moderate bilateral disease; some postural instability; physically independent; 4 Severe disability; still able to walk or stand unassisted, 5: Wheelchair bound or bedridden unless aided

**Supplementary References**

Poletti, M. et al., (2013). The relationship between motor symptom lateralization and cognitive performance in newly diagnosed drug-naïve patients with Parkinson’s disease. *Journal of Clinical and Experimental Neuropsychology*, *35*(2), 124–131.
